# Validation of Whole Blood Rapid Diagnosis Test for Hepatitis B

**DOI:** 10.5334/aogh.2866

**Published:** 2020-05-21

**Authors:** Jose D. Debes, Gertine van Oord, Andre Boonstra

**Affiliations:** 1 Department of Medicine, University of Minnesota, Minneapolis, MN, US; 2 Department of Gastroenterology and Hepatology, Erasmus MC, Rotterdam, NL

## Abstract

We recently published a report validating point-of-care rapid diagnostic tests (RDT) for the diagnosis of Hepatitis B virus (HBV) infection in serum. In the current report, we validated a whole-blood RDT for HBsAg in the form of a test-strip. The test was validated in 55 HBV positive individuals across all genotypes other than F, and in 20 HBV negative individuals in the Netherlands. The RDT showed 100% sensitivity and specificity. The low cost and use in whole blood allows this RDT to be useful in resource-limited locations, further validation in such settings will be of importance.

To the editor:

We recently published a report validating point-of-care rapid diagnostic tests (RDT) for the diagnosis of Hepatitis B virus (HBV) infection [1]. We believe that expedited and cost-effective diagnosis of HBV infection via RDT is critical to understand the epidemiology of HBV in resource-limited settings and implement prevention and treatment strategies [2]. In that report, a whole-blood rapid test for HBV surface antigen (HBsAg), the universally accepted test to diagnose chronic HBV infection, in the form of “cassette” did not produce reliable results, with a sensitivity of only 56% (albeit a specificity of 100%). In the current report, we validated a whole-blood RDT for HBsAg in the form of a test-strip (PRECHECK Bio Inc, Korea). The test was validated in 55 HBV positive (HBV-pos) and 20 HBV negative (HBV-neg) individuals in the Netherlands. The median age was 38 years (IQR 3–47) in the HBV-pos group, with 60% being male and 33 years (IQR 30–37) in the HBV-neg group, with 85% being male. The RDT showed 100% sensitivity and specificity, with complete correlation for HBsAg positive and negative results with the local gold standard at Erasmus University, Rotterdam (LIAISON XL, Diaorin, Italy). All HBV-pos individuals were negative for hepatitis C and D virus, as well as human immunodeficiency virus. The samples included individuals on treatment and inactive carriers, all HBV genotypes other than F, with the most common genotypes being D (23%), C (20%), and A (15%). Median HBV DNA was 20 IU/ml (IQR 20–285), median HBsAg level 510 IU/ml (IQR 150–2900), and 16 subjects (29%) were HBeAg positive. Seventy percent of HBV+ individuals were on treatment (47% on entecavir and 21% on tenofovir).

We believe this addition to our previous study is important as it provides validation of a RDT for HBsAg using whole blood, in a rapid and cost-effective manner, therefore allowing the test to be used without the need of any laboratory resources. Our test was validated in one institution (Erasmus MC, the Netherlands) in a variety of genotypes. However, we believe further validation in resource-constrained settings will be of importance.

## Data Accessibility Statement

Study data will be made available upon request of the corresponding author.
